# A rare paediatric mandibular desmoid tumour: emphasis on growth-conscious resection and reconstructive strategy

**DOI:** 10.1093/jscr/rjaf526

**Published:** 2025-07-14

**Authors:** Amine Oussalem, Walter J J Bahizi, Bouchra Dani, Malik Boulaadas

**Affiliations:** Department of Maxillo-Facial Surgery, Specialty Hospital, Area Lamfadel Cherkaoui, Rabat - Institut, Rabat B.P 6527, Morocco; Department of Maxillo-Facial Surgery, Specialty Hospital, Area Lamfadel Cherkaoui, Rabat - Institut, Rabat B.P 6527, Morocco; Department of Maxillo-Facial Surgery, Specialty Hospital, Area Lamfadel Cherkaoui, Rabat - Institut, Rabat B.P 6527, Morocco; Department of Maxillo-Facial Surgery, Specialty Hospital, Area Lamfadel Cherkaoui, Rabat - Institut, Rabat B.P 6527, Morocco

**Keywords:** desmoid tumour, aggressive fibromatosis, mandible, paediatric oncology, jaw neoplasms

## Abstract

Desmoid-type fibromatosis (DTF) of the mandible is a rare, benign histological tumour that behaves aggressively in paediatric patients. We report the case of a 15-year-old boy with a 1-year history of a painless mandibular mass resulting in facial asymmetry. Imaging revealed a large, well-defined lesion of the right mandibular ramus and angle. Incisional biopsy confirmed DTF. Surgical resection was performed via hemimandibulectomy with preservation of the condyle. Immediate reconstruction was achieved using a titanium plate, preserving both facial aesthetics and oral function. Histopathological analysis confirmed complete excision with tumour-free margins. No recurrence was observed at the 12-month follow-up. This case highlights the importance of a growth-sensitive surgical strategy and multidisciplinary collaboration in managing paediatric mandibular DTF.

## Introduction

Desmoid-type fibromatosis (DTF) is a rare, benign fibroblastic tumour with intermediate biological behaviour and no metastatic potential [1]. It accounts for ˂3% of all soft-tissue neoplasms and primarily affects adolescents and young adults [2]. Although DTF most commonly arises in the abdominal wall, extra-abdominal locations—including the head and neck—are well documented [3]. Mandibular involvement remains exceptional, particularly in children, and poses specific diagnostic and therapeutic challenges [4].

Clinically, DTF often presents as a slowly enlarging, painless mass. Imaging findings are often non-specific, making histopathological confirmation essential for diagnosis [5]. Despite its benign nature, DTF is locally infiltrative and associated with a high risk of recurrence [6].

Surgical excision with negative margins remains the mainstay of treatment, though in paediatric patients, achieving optimal resection requires balancing oncologic control with the preservation of growth, function, and aesthetics [7]. We report a rare case of paediatric mandibular DTF managed with segmental resection and immediate reconstructive plating, emphasizing growth-conscious surgical decision-making.

## Case report

**Figure 1:**
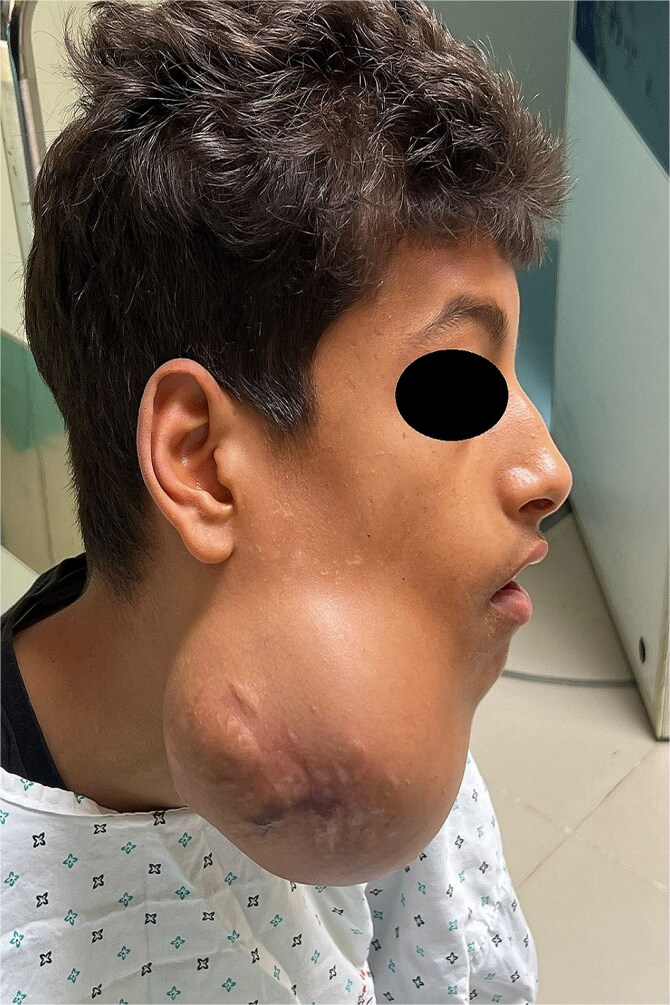
Preoperative lateral view showing the swelling of the right hemiface caused by the mandibular tumour.

**Figure 2:**
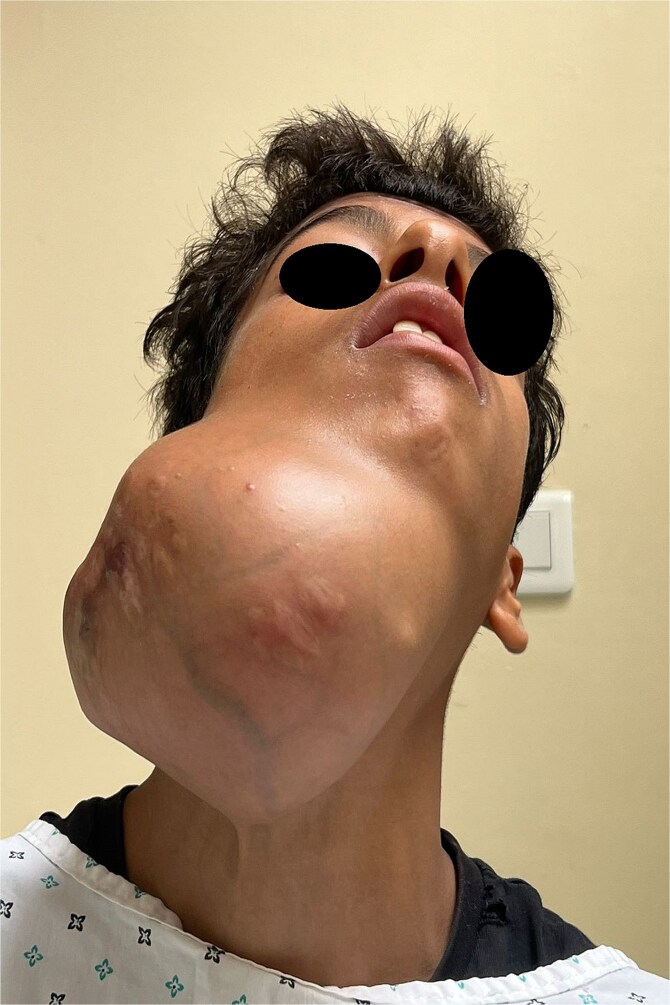
Frontal view illustrating facial asymmetry due to the massive mandibular tumour.

A 15-year-old boy with no significant medical history presented with a painless, progressively enlarging swelling of the right mandibular region evolving over 1 year and causing facial asymmetry. Clinical examination revealed a firm, immobile, non-tender mass at the right mandibular angle and ramus. Intraorally, molar displacement was noted, without trismus or mucosal involvement. Sensory function was preserved (Figs 1 and 2).

**Figure 3:**
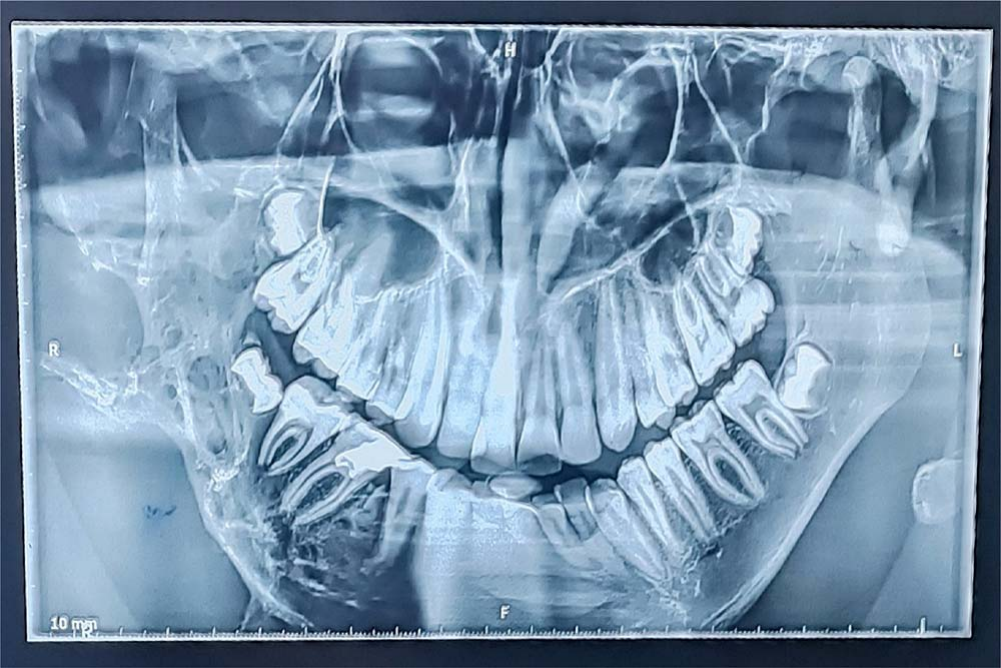
Panoramic radiograph (OPT) revealing an extensive osteolytic lesion of the right hemimandible.

**Figure 4:**
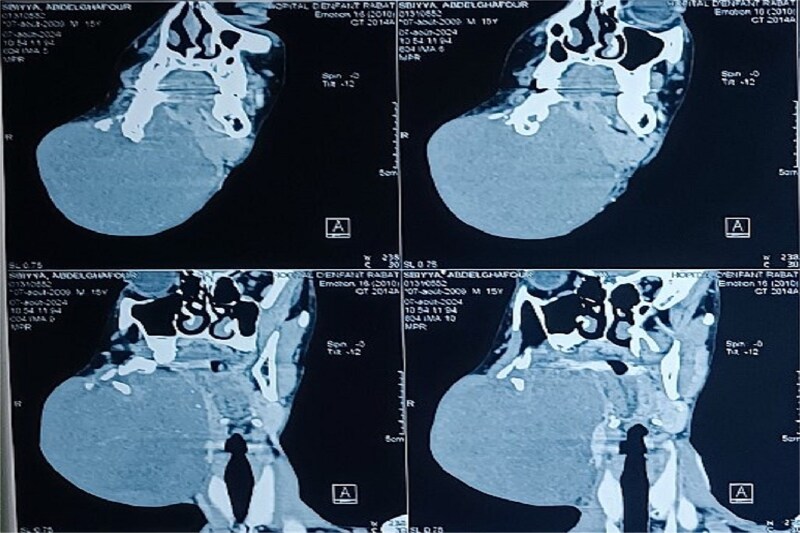
Coronal CT scan in soft tissue window demonstrating a well-defined osteolytic mandibular lesion with cortical thinning.

Panoramic radiography showed a well-defined radiolucent lesion involving the right mandibular angle and ramus. Coronal computed tomography (CT) scan in soft tissue window demonstrating a well-defined osteolytic mandibular lesion (12 × 9 × 14 cm) with cortical thinning (Figs 3 and 4).

An intraoral incisional biopsy showed spindle-shaped fibroblasts in sweeping fascicles with infiltrative borders, low mitotic activity, and no atypia. Immunohistochemistry was positive for β-catenin, consistent with DTF.

**Figure 5:**
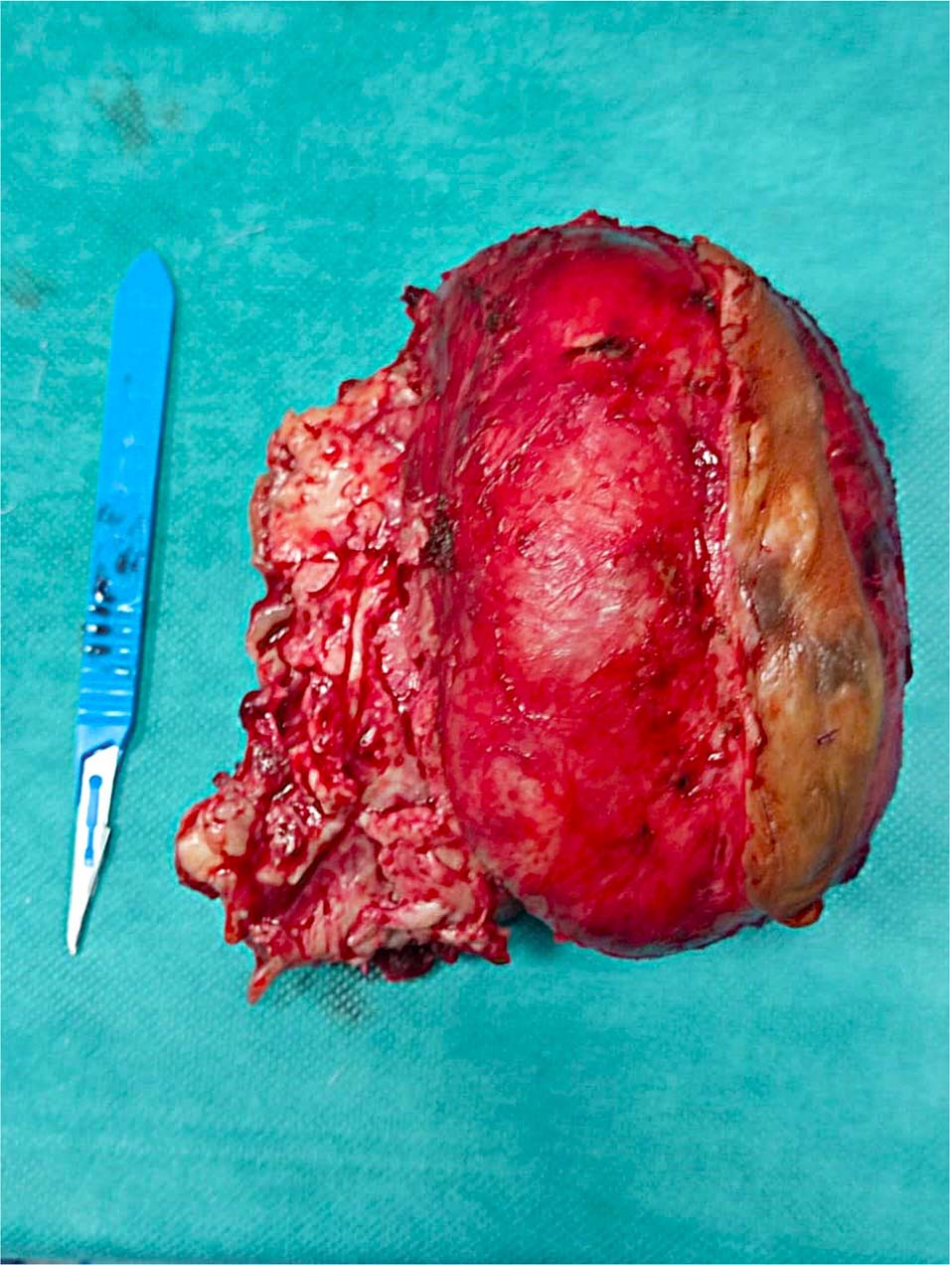
Gross specimen of the resected tumour, displaying its macroscopic features.

A right hemimandibulectomy sparing the condyle was performed via submandibular approach. Clear margins (≥1 cm) were obtained, and immediate reconstruction with a pre-contoured titanium plate was achieved. Recovery was uneventful. Final pathology confirmed complete excision (Fig. 5).

**Figure 6:**
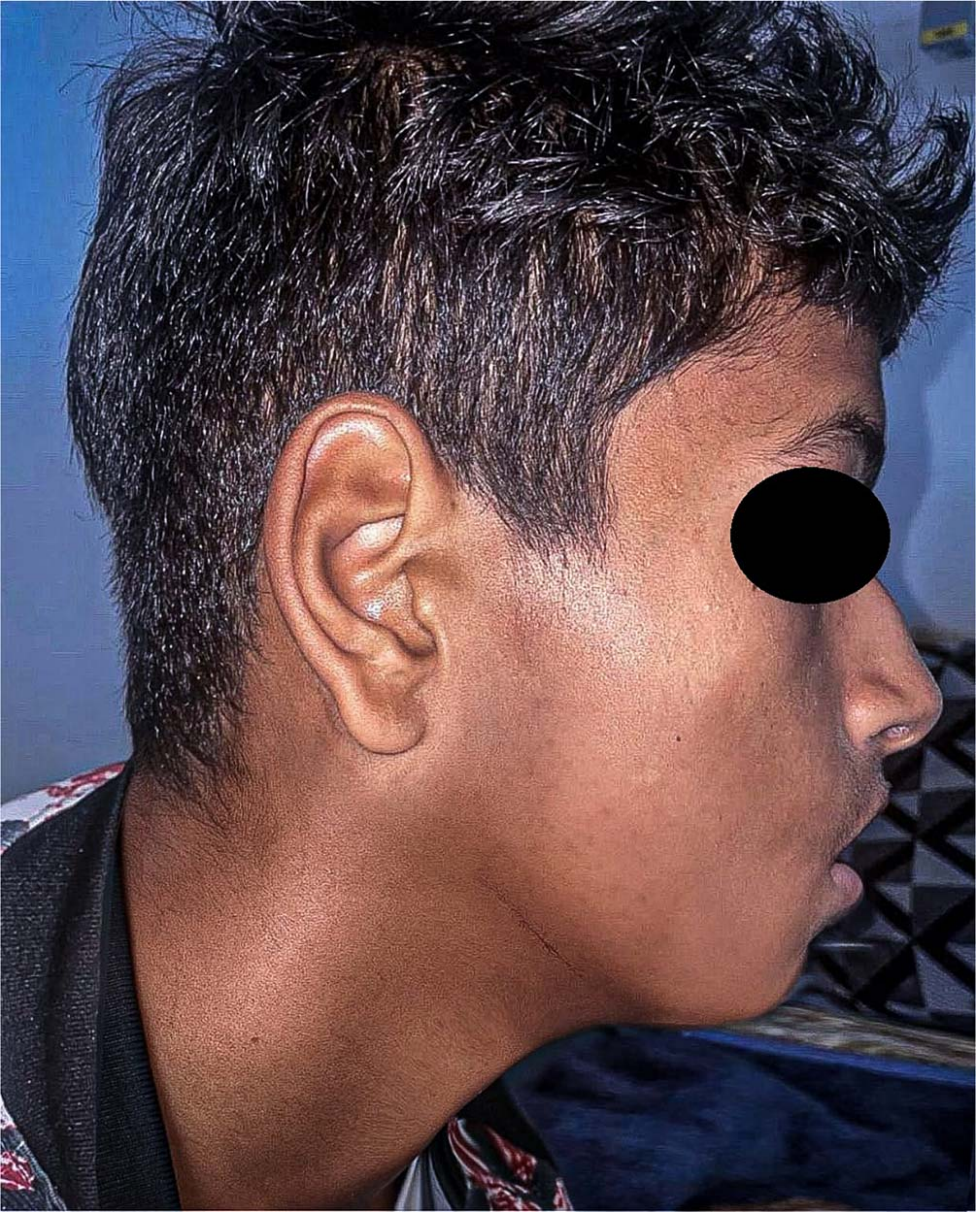
Postoperative photograph of the patient at 1-year follow-up.

At 12-month follow-up, the patient showed no recurrence, preserved function, and satisfactory facial aesthetics (Fig. 6).

## Discussion

DTF of the mandible is extremely rare in paediatric patients, accounting for ˂0.03% of head and neck tumours [1]. Although histologically benign, DTF is locally aggressive with a high recurrence rate, especially in children. Its slow growth often delays diagnosis, and patients usually present at an advanced stage.

Imaging is essential for assessing bone destruction and soft tissue extension. CT typically shows well-defined osteolytic lesions with cortical thinning, whilst MRI offers better soft-tissue characterization, revealing intermediate to high T2 signal intensity [2, 3]. However, imaging remains non-specific, and histopathological confirmation is necessary, with nuclear β-catenin expression being a key diagnostic marker [4].

Radical surgical resection with negative margins remains the mainstay of treatment, as incomplete excision is linked to recurrence [5]. In children, however, surgical planning must balance oncologic control with preservation of mandibular growth, aesthetics, and function. Mandibular resection may impair dentofacial development, occlusion, and joint mechanics [6].

In our case, extensive tumour involvement required a right hemimandibulectomy. Immediate reconstruction with a titanium reconstruction plate was performed. Although vascularized bone grafts like fibula free flaps allow mandibular continuity and dental rehabilitation, they are often deferred in younger children to minimize donor site morbidity and avoid interfering with skeletal growth [7, 8]. In adolescents nearing maturity, free flaps may be considered, but the choice should be tailored to age, anatomy, and overall status.

Reconstruction plates offer immediate continuity with low morbidity but do not support future growth. Secondary bone grafting is often needed at skeletal maturity to enable dental rehabilitation and restore function [9]. Long-term follow-up is essential to monitor symmetry, joint function, and the need for further surgery.

Early physiotherapy is crucial to preserve jaw mobility and prevent trismus or ankylosis. Our patient began rehabilitation 3 weeks postoperatively. Psychological support is also important to address body image concerns and promote emotional adjustment.

Adjuvant treatments like radiotherapy or chemotherapy are generally avoided in children unless the tumour is unresectable or recurrent [10]. Molecular therapies targeting the Wnt/β-catenin pathway are still investigational but may offer options in refractory cases [11].

At 1 year, the patient remained recurrence-free, with good function and aesthetics. Nonetheless, extended follow-up beyond skeletal maturity is critical to detect late recurrence and assess the timing and modality of definitive bone reconstruction.

This case contributes to the scarce literature on paediatric mandibular DTF and underscores the value of a growth-conscious, multidisciplinary approach prioritizing both oncologic control and developmental outcomes.

## Conclusion

Mandibular DTF in paediatric patients remains a rare but challenging entity due to its aggressive local behaviour and recurrence risk. Early diagnosis, radical resection with clear margins, and structured postoperative care are key to successful outcomes. This case highlights the importance of a multidisciplinary approach and long-term follow-up to optimize both oncological and functional results.
